# High Resolution Spatial Mapping of Human Footprint across Antarctica and Its Implications for the Strategic Conservation of Avifauna

**DOI:** 10.1371/journal.pone.0168280

**Published:** 2017-01-13

**Authors:** Luis R. Pertierra, Kevin A. Hughes, Greta C. Vega, Miguel Á. Olalla-Tárraga

**Affiliations:** 1 Area de Biodiversidad y Conservación, Universidad Rey Juan Carlos, Móstoles, Spain; 2 British Antarctic Survey, National Environment Research Council, High Cross, Madingley Road, Cambridge, United Kingdom; Friedrich-Schiller-Universitat Jena, GERMANY

## Abstract

Human footprint models allow visualization of human spatial pressure across the globe. Up until now, Antarctica has been omitted from global footprint models, due possibly to the lack of a permanent human population and poor accessibility to necessary datasets. Yet Antarctic ecosystems face increasing cumulative impacts from the expanding tourism industry and national Antarctic operator activities, the management of which could be improved with footprint assessment tools. Moreover, Antarctic ecosystem dynamics could be modelled to incorporate human drivers. Here we present the first model of estimated human footprint across predominantly ice-free areas of Antarctica. To facilitate integration into global models, the Antarctic model was created using methodologies applied elsewhere with land use, density and accessibility features incorporated. Results showed that human pressure is clustered predominantly in the Antarctic Peninsula, southern Victoria Land and several areas of East Antarctica. To demonstrate the practical application of the footprint model, it was used to investigate the potential threat to Antarctica’s avifauna by local human activities. Relative footprint values were recorded for all 204 of Antarctica’s Important Bird Areas (IBAs) identified by BirdLife International and the Scientific Committee on Antarctic Research (SCAR). Results indicated that formal protection of avifauna under the Antarctic Treaty System has been unsystematic and is lacking for penguin and flying bird species in some of the IBAs most vulnerable to human activity and impact. More generally, it is hoped that use of this human footprint model may help Antarctic Treaty Consultative Meeting policy makers in their decision making concerning avifauna protection and other issues including cumulative impacts, environmental monitoring, non-native species and terrestrial area protection.

## Introduction

Antarctica, taken to be the area south of latitude 60°S, is increasingly subject to conservation challenges, including the impacts of climate change, habitat disturbance and destruction, non-native species introductions and increasing biological homogenization resulting from increasing inter-regional connectivity [1, 2, 3, 4]. Terrestrial habitats, in particular, are under increasing human pressure [5, 6]. Circa 99.8% of the continent’s 14,000,000 km^2^ area is covered with permanent ice or snow [7] and, of this, only c 6,000 km^2^ is found within 2 km of the coast and therefore suitable for the establishment of research stations that can be relatively easily resupplied by ship [8]. However, this is also the area that supports the majority of Antarctica’s macroscopic terrestrial life and provides breeding sites for bird populations. Inevitably this has led to the competing uses of suitable ice-free ground for the establishment of human infrastructure (including airstrips, wharfs, laboratories and living accommodation) versus the conservation of relatively flat coastal ice-free ground for increasingly scare terrestrial habitats [9, 10, 11, 12]. As a result, Antarctica’s bird populations may be particularly vulnerable to local human activities such as visitor disturbance and aircraft over flight, in addition to global environmental impacts, such as climate change, which may affect habitats and food availability [13, 14, 15, 16, 17].

Human footprint models provide important insights into the influence of humans within ecosystems [18, 19]. They can act as a relevant predictor for biogeographic processes and, as a consequence, can be an important consideration in Species Distribution Models (SDMs) [20]. Human footprint modelling has been undertaken across the globe (e.g. [21]) but, until now, Antarctica has been largely omitted from global maps [22], possibly because there is no native human population and inhabitants are transient national Antarctic programme science and support staff [23]. Nevertheless, up to c. 4000 people from national programmes and 40,000 tourists and tourism industry staff visit the continent annually [24, 25]. In addition, information on human presence and activities may be difficult to compile as over 30 nations are active in the region, each with varying amounts of permanent infrastructure distributed over different areas of the continent [24] and with highly variable formats for national reporting on activities conducted by operators (see for instance [26] on permit allocation, and [27] on biosecurity).

As a result only a few regional or local attempts to measure human footprint in Antarctica have been undertaken. A recent study [28] mapped the footprint of the British national Antarctic programme (the British Antarctic Survey; BAS) over the previous 70 years, in terms of infrastructure location and expedition visitation across the Antarctic Peninsula region, and observed substantial spatial and temporal variability. At a more local scale, another study [29] detailed the footprint of a scientific camp across Antarctic Specially Protected Area (ASPA) No. 126 at Byers Peninsula, South Shetland Islands, which protects lake ecosystems and terrestrial communities. Chown and colleagues [1] evaluated the risk of biological invasions to the continent by mapping areas of human settlement and visitation and environments with climates suitable for potential invaders. Such locations could be also vulnerable to the introduction of disease microorganism [30]. The work of Chown and colleagues [1] indirectly provided a first insight on the extent of human footprint across the continent; however, the pressure metric applied was based purely on human densities, and related to other variables specifically directed to estimate the biological risk of non-native species establishment. Furthermore, the risk map generated was limited to a spatial resolution of 50 x 50 km. Other authors have displayed the distribution of Antarctic facilities [23], quantified tourism industry vessel routes around the Antarctic Peninsula [31, 32] and examined levels of visitation to protected areas [26, 33]. Collectively, these studies reveal that substantialt parts of ice-free Antarctica are not currently free from human activities and resulting impacts, making a precise view of current spatial pressure, which has resulted from past temporary or permanent activities, essential for local and continent-wide policy development and management action [34, 35, 36].

In this work, we assimilated available datasets and reproduced the approach of Sanderson and colleagues [21] to generate a high-resolution multidimensional formula showing the comparative spatial human footprint across Antarctica at a small scale (30 arcsec). This system integrated all recurrent elements, i.e. land use, density and accessibility dimensions, that would complete and complement the latest global mapping [22] but taking into consideration to the special particularities of the Antarctic continent. Recently, 204 Antarctic Important Bird Areas (IBAs) were identified by BirdLife International and the Scientific Committee on Antarctic Research (SCAR) [3] and subsequently acknowledged by the Antarctic Treaty Consultative Meeting (the body which governs Antarctica) as potentially useful for environmental impact assessments, monitoring and protected area planning (Resolution 5 (2015)). To demonstrate a potential application of the footprint model we incorporated the Antarctic IBA data to investigate the vulnerability of bird populations to local human activities and considered the level of protection currently afforded to these areas under the Antarctic Treaty System.

## Materials and Methods

In this study, human footprint was considered to be the spatial pressure on Antarctic ice-free ground, caused either by the existing (i.e. currently operating facilities) or potential presence (in terms of accessibility) of any human activity within the continent and off-shore islands located south of latitude 60°S. Footprint mapping was undertaken using ArcGIS 10.0 software with contour layers of Antartica from the Antarctic Digital Database–Version 6. Five spatial features relating to different human capabilities were aggregated in the development of the Antarctic footprint model. Every ice-free pixel covered 30 arcseconds, which is equal to 0.0083333 degrees (representing 1 km x 1 km at the equator, and a decreased longitude in Antarctica, following a trigonometric cosine-based fashion) under the WGS84 coordinate system. This system aimed to retain geodesic pixel size and directly match standards for world scale analyses [22]. Each pixel had assigned a score ranging from 1 to 10 per feature based on the following rules (see below). It should be noted that in all features when one pixel fell to more than one category of the same feature the highest score among them was assigned.

### Feature 1: land use

A set of ground rules were applied to generate six land use categories:

**Built environments for antarctic stations**: point locations of all facilities on ice-free ground listed by the Council of Managers of National Antarctic Programs (COMNAP) [24], and updated within the Antarctic Digital Database (ADD), were scored as ‘built environment’ with a radius of 500 m with a value of 10 out of 10.

**Stations’ area of influence:** the area considered to be influenced by scientific, recreational and logistical activities from such facilities was all ice-free ground within a 5 km radius (including all inmediately surrounding ice-free ground accessible by boat or over-snow transportation, e.g. snow mobile) and given a value of 6 out of 10. This area of influence aimed to reflect all ground that was recurrently trampled around stations due to the existence of nearby research sites of interest and/or the deployment of remote sensing stations that required regular maintenance, as well as ground subject to walks and other recreational activities. Smaller facilities, including all camps, refuges, aerodromes and small stations accommodating fewer than 10 people, or where no information was available, were placed into another land cover feature category with a built-up area of radius 500 m and given a score of 8 out of 10.

**Visitor landing sites:** the coordinates supplied by the International Association of Antarctica Tour Operators (IAATO) for all designed visitor sites within the Antarctic Peninsula were considered as landing sites with a radius of 500 m and given a value of 9 out of 10 for each site to reflect potential land transformation.

**Visitor sites’ area of influence:** an area of influence of radius 2.5 km was generated for all visitor sites to account for visitor movement around the visitor site point locations. These sites were given a value of 5 out of 10.

**Protected areas:** entry to each Antarctic Specially Protected Area (ASPA) is allowed only in accordance with a permit provided by an appropriate national authority (typically a government agency) and, consequently, these protected areas were considered as a separate category in the model. ASPA land cover was extracted from available satellite-derived British Antarctic Survey protected areas data (see [37]) or from the relevant ASPA Management Plans (available from: http://www.ats.aq/devPH/apa/ep_protected.aspx?lang=e). A score of 3 out of 10 was given to these areas. Antarctic Specially Management Areas, which are designated to help facilitate co-ordination of human activities within an area, were not incorporated as they generally overlapped with high score categories.

**Remote sites:** remaining ice-free areas were classified as remote sites, and given a score of 1 out of 10.

The scoring system for each land cover feature was chosen based on ‘land transformation’ in [21], but adapted to Antarctic particularities, being heavily influenced by literature concerning environmental impacts in Antarctica from local human activities (S1 Table) [5].

### Feature 2: human density

For human density the reference unit used is inhabitants’ km^-2^ y^1^ ranging from 1 to 10 (for 10 or more inhabitants km^-2^ y^1^) in order to match the scoring determined by Sanderson and colleagues [21] (see S2 Table). Although there are no inhabitants living permanently in Antarctica we generated this index by the addition of likely individual periods of residence or visitation:

**‘Facilities’ (stations or camps):** we attributed a 50% average occupancy of their maximum capacity. For stations operating only seasonally and seasonal camps, we corrected this value by incorporating an estimated operational period duration of four months and two months, respectively. Therefore, the formulae were:

$$Year-round\;facilities:\;Density=\frac{Max.Capacity\;\times 0.5}{1\;(Twelve\;Active\;Months)}$$

$$Seasonal\;stations:\;Density=\frac{Max.Capacity\;\times \;0.5\;}{3\;(Four\;Active\;Months)}$$

$$Seasonal\;camps:\;Density=\frac{Max.Capacity\;\times \;0.5\;}{6\;(Two\;Active\;Months)}$$

Stations that received tourist visits had these ‘inhabitant’ values added to their human density scores following the formula described below.

**‘Landing sites’ of tourist operators:** we considered that the sum of three individual landings per day corresponded to 1 inhabitant per day. Therefore the formula was:

$$Inhabitants\;y-1=\frac{Visitor\;Numbers\;}{3\;(Landings\;Per\;Day)\;\times \;365\;(Days\;of\;the\;Year)}$$

As a reference, the maximum density can be estimated using the following assumptions: in general, and taking into consideration limits within the Site Guidelines for Visitors (see: http://www.ats.aq/e/ats_other_siteguidelines.htm), each visitor site can typically accommodate c. 150 visitors per landing, with 3 landings per day = 150 inhabitants per day, if we assume a maximum active season of 200 days (a recent study [32] found that the longest tourist season recorded so far was 175 days in 2008–09) the maximum theoretical density will be 82 inhabitants y^-1^ km^-2^ at a landing site.

**Protected areas’:** visitation density was assessed based upon the number of people issued with permits for ASPA entry (taken from [26]) with an estimated length of 20 days of visitation and consideration of the ASPA size. Therefore, the formula was:

$$ASPA\;Density=\frac{Permits\;per\;sq\;km\;}{365\;(Days\;of\;the\;Year)}\;\times \;20\;(Active\;Days)$$

### Feature 3: accessibility

Three separate dimensions were considered during the calculation of accessibility to ice-free areas, with their corresponding scores accounted separately: (1) coastal access from shipping, (2) terrestrial access from facilities and (3) airborne access from aerodromes. Inclusion of existing terrestrial routes was considered for the calculations, but due to the absence of centralized information on paths, tracks and roads this feature was neglected; thus we acknowledge a limitation within the study to establish the exact routes of overland movement.

‘Coastal access’ accounts for all ice-free grounds that can be accessed by ship landings. The scoring system was created using a radial buffer zoning based on walking distances from the nearest coast (1, 2, 4, 8 km, and so on…). All ice-free areas separated from coasts were given a value of 1.

When calculating the ‘Access from facilities’ dimension, the distance to the nearest facility (e.g. station, camp, or refuge) was considered. The scoring system was also created using radial buffer zoning (see S3 Table). For example, a value of 5 was given to a radius zone of 16–32 km, which was considered to be a maximum walkable distance per day. In turn, longer distances are still able to be covered by land based helicopter operations (with >256 km scored as 1).

When considering ‘Airborne access’, flight distance from reported aerodromes was added as another feature to the human footprint calculations. Taking into consideration the flight endurance of a typically employed small aircraft (such as a DeHavilland Twin Otter or Dornier 228) and allowing for return trips, ice-free ground within a radius of 450 km of each airstrip was considered within normal operational range for land based aircraft operations within Antarctica. It was noted that greater distances may be covered by larger aircraft (such as the Lockheed C130 Hercules, Lockheed C-5 Galaxy or Boeing C-17) but flights greater than 450 km were not differentiated here (all scored as 1). In turn, movement of helicopters from ships were not quantified, thus their ship-based capabilities were only partially assessed within the coastal accessibility dimension.

### Aggregated human footprint values per site

To obtain the aggregated human footprint score for each ice free site, the values from the five features analyzed within the Antarctic continent were added and obtained scores ranging from 5 to 50. Data were then re-scaled to the range 1 to 100 to allow direct comparison with maps created for other parts of the world and thereby allow integration with spatial modeling datasets outside Antarctica [21, 22].

In addition, the scores for large scale infrastructure on permanent ice (e.g. Amundsen-Scott South Pole Station, Vostok Station, Concordia Station, Neumeyer III Station, Halley VI Research Station, Kunlun Station) (see S4 Table) and the IBAs on ice (see description below) were also calculated to allow for comparisons by applying the previous formulas to their point locations

#### Human footprint values for antarctic important bird areas

Harris et al. (2015), in association with BirdLife International, generated an authoritive and comprehensive report on bird populations within Antarctica and identified those areas most important for birds (Important Bird Areas; IBAs). The report reviewed available and historic information on different bird species populations throughout Antarctica and generated a list of 204 IBAs. We used information contained within this report as a basis for our work to demonstrate a potential application of the footprint model to investigate the potential vulnerability of bird populations to local human pressure.

The list and location of Antarctica’s 204 Important Bird Areas (IBAs) was obtained from [3]. The human footprint value for each IBA point location was obtained using the footprint model described in this paper. Values for bird colonies within IBAs located on ice, such as emperor penguin colonies, were manually calculated following the principles described earlier. A score of 5 was given to ‘coastal accessibility’ in order to reflect their 8–16 km average distance to open water and account for seasonal variability in sea ice conditions. The ‘land use’ score was fixed to zero as a distinct category (ice) and ‘human density’ to 1 (1 or less inhabitants km^-2^ y^1^). Therefore, airborne/station accessibility largely determined variability in footprint values between IBAs located on ice.

We calculated the percentage of Antarctic bird species estimated global populations (breeding pairs) contained within IBAs that were also afforded legal protection through their designation as Antarctic Specially Protected Areas (ASPAs). The estimated global populations (breeding pairs) of bird species were obtained from information collated by [5] from a range of literature sources [9, 38, 39, 40, 41, 42, 43]. Bird population numbers within each IBA were also derived from [5] where the original source references for the available population data can be seen for each species within each IBA. Within IBAs substantial variability in bird population counts may have been recorded in different years, so in this assessment the most recent counts were generally used.

## Results

### Antarctic human footprint distribution

**Fig 1:**
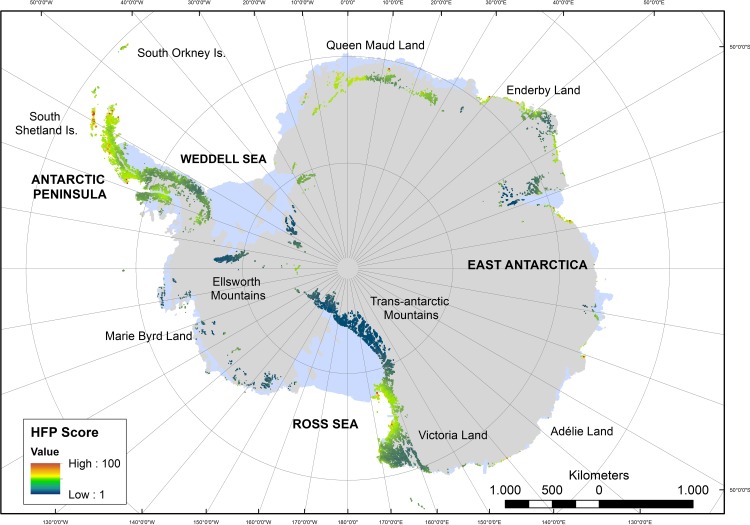
Human footprint of the Antarctic continent and offshore islands (for inset, see Fig 2).

Modelling of the current distribution of human activities in Antarctica revealed a footprint that was largely concentrated in the northern Antarctic Peninsula region and southern Victoria Land, as well as several isolated and predominantly coastal areas in East Antarctica (Fig 1 and S4 Table). Compared to other areas of the world, large expanses of Antarctica remain relatively unvisited by humans, including much of Marie Byrd Land and inland areas such as the Transantarctic, Ellsworth and Prince Charles Mountains.

**Fig 2:**
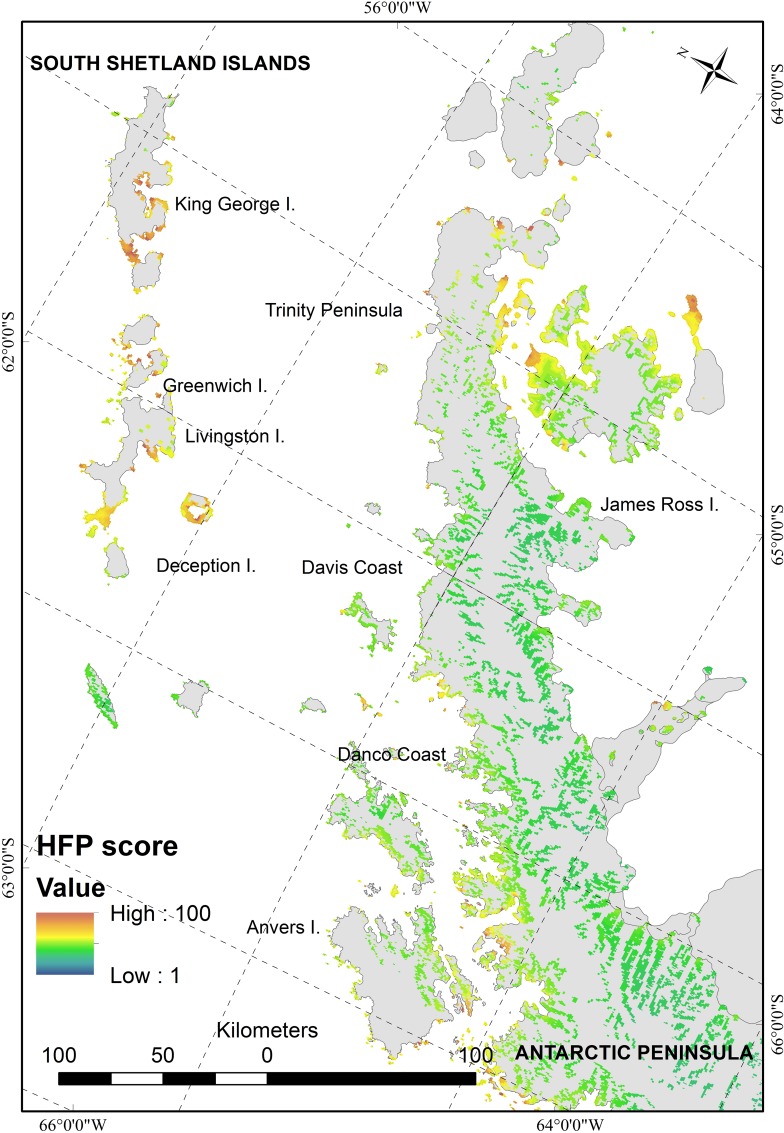
Human footprint map of the South Shetland Islands and northern Antarctic Peninsula.

The Antarctic Peninsula scored some of the highest footprint values, and in particular the ice-free areas of the South Shetland Islands and northern Antarctic Peninsula, where numerous stations and visitor sites are concentrated (Fig 2). Notably, the vicinity of Frei, Escudero, Bellinghausen and Great Wall stations on Fildes Peninsula (King George Island, South Shetland Islands) displays the largest cluster of pixels in the Antarctic Peninsula region with a footprint score of over 90 across an area of c. 4.5 km^2^. Human footprint scores remained moderately high along the western side of the Antarctic Peninsula but declined south of latitude 67°S. In contrast, footprint values on the eastern side of the Antarctic Peninsula was substantial only at latitudes above c. 65°S. Southern Victoria Land had high scores in the area of McMurdo Sound where McMurdo Station (US; Antarctica’s largest research station) and Scott Base (New Zealand) are located. In general, ASPAs were subject to less footprint pressure and individual ASPAs generally scored the equivalent of fewer than 1 inhabitant y^-1^ except within a small subset of particularly highly frequented ice-free ASPAs within the South Shetland Islands and the ASPAs designated to protect the historic huts of Victoria Land.

### Antarctic important bird areas

**Fig 3:**
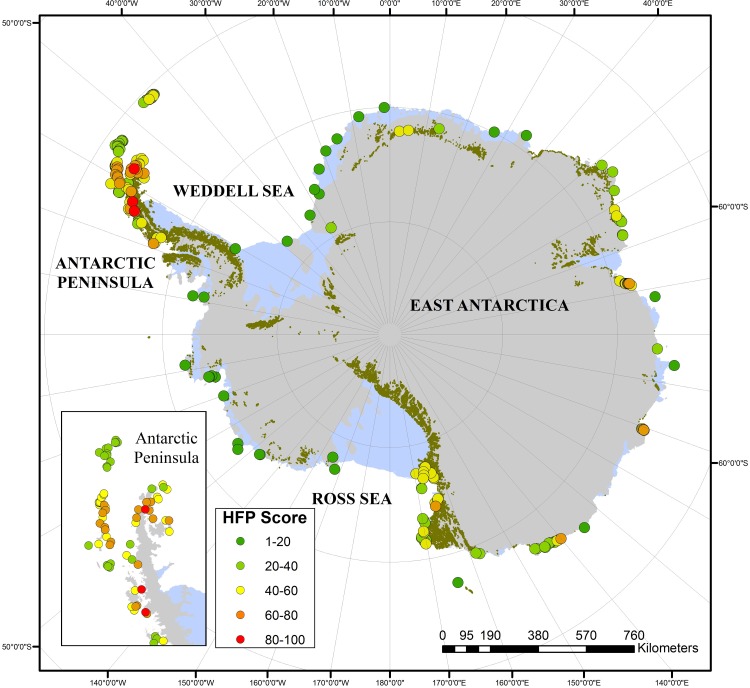
Map of Antarctica showing the distribution of the continent’s Important Bird Area (IBAs) and the human footprint (HFP) scores (low score: low human footprint; high score: high human footprint).

**Fig 4:**
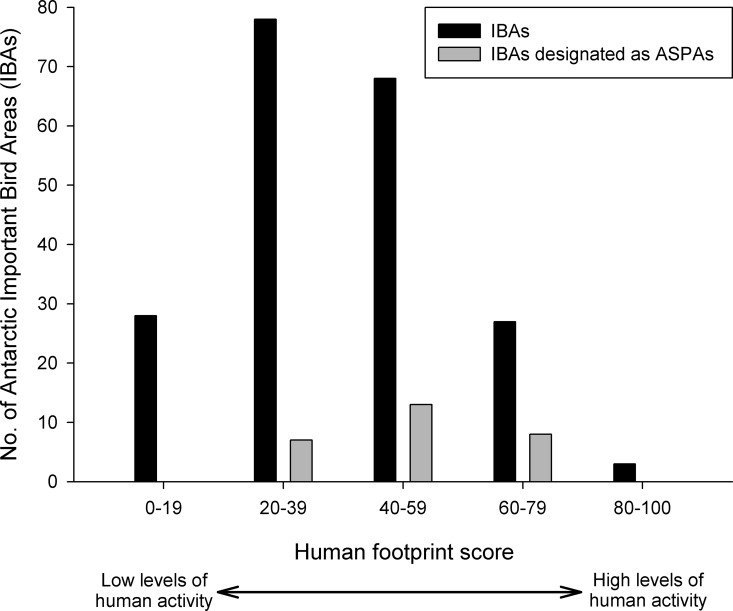
Human footprint scores for Antarctic Important Bird Areas (IBAs).

Fig 3 shows the distribution of human footprint values across all 204 IBAs, with colonies subject to the highest human footprint located predominantly around the northern Antarctic Peninsula. Table 1 shows the 10 most potentially vulnerable IBAs based upon human footprint values and also details the triggers for the allocation of IBA status and the area management tools employed by the Antarctic Treaty System. Some IBAs are located in close proximity to established research stations and visitors sites, resulting in a high footprint value. However, some areas identified as potentially vulnerable to local human activity have no formal internationally agreed area management (e.g. IBA No. ANT074: Hope Bay, northern Antarctic Peninsula). Fewer than 14% of the 204 IBAs have been afford legal protection through designation as Antarctic Specially Protected Areas (28 ASPAs; see Table 2), but only eight of the IBAs with ASPA status had relatively high human footprint scores (i.e. >60) and therefore are potentially more at risk from local human activity (see Fig 4). To see if the current ASPA system was effective in affording legal protection to Antarctica’s bird populations, we attempted to determine the percentage of the estimated global population of Antarctic bird species found within Antarctic IBAs that have also been designated as ASPAs (Table 3). Substantial variability in the percentage of bird species protection within ASPAs was noted. Some species (including south polar skuas and chinstrap, Adélie and emperor penguins) had more than 10% of their populations breeding within ASPAs; in contrast, many smaller species (e.g. some terns and petrels) were apparently less well protected, with < 1% of the global population breeding within ASPAs, although the data for these species may be much less accuarate, due to colony remoteness and/or counting difficulties, making interpretation difficult. Insufficient data was available to make an assessment for some species, including the macaroni penguin (*Eudyptes chrysolophus*), which has been categorized as Vulnerable by the IUCN (Table 3).

**Table 1:** Antarctic Important Bird Areas (IBAs) subject to the highest levels of estimated human footprint (10 highest footprint scores).

No.	Antarctic Important Bird Area	Region	Trigger species (IBA criteria)^1^	HFP Score	Main human footprint source	Site Guidelines for Visitors^2^	Antarctic Specially Protected Area (ASPA)^3^	Antarctic Specially Managed Area (ASMA)^4^
1	ANT074: Hope Bay	Trinity Peninsula	Adélie Penguin (A1, A4ii) Adélie Penguin (A4iii)	97	Esperanza and Ruperto Elichiribehety Stations			
2	ANT089: Petermann Island	Graham Coast	Gentoo Penguin (A1, A4ii)	84	Popular tourist site	✓		
3	ANT083: Cuverville Island	Palmer Archipelago, Danco Coast	Gentoo Penguin (A1, A4ii)	82	Popular tourist site	✓		
4	ANT073: Brown Bluff	Trinity Peninsula	Adélie Penguin (A4iii)	78	Popular tourist site	✓		
5	ANT048: Ardley Island, King George Island	South Shetland Islands	Gentoo Penguin (A1, A4ii)	75	Frei, Escudero, Bellinghausen and Great Wall Stations; popular tourist site	✓	ASPA No. 150	
6	ANT081: Cierva Point and offshore islands	Palmer Archipelago, Danco Coast	South Polar Skua (A4ii)	71	Primaver Base		ASPA No. 134	
7	ANT085: Cormorant Island	Palmer Archipelago, Danco Coast	Imperial Shag (A4i)	71	Palmer Station			ASMA No. 7 (Restricted Zone)
8	ANT045: Point Hennequin, King George Island	South Shetland Islands	South Polar Skua (A4ii)	69	Vicente, Feraz, Machu Picchu and Arctowski Stations			ASMA No. 1 (Scientific Zone)
9	ANT047: Potter Peninsula, King George Island	South Shetland Islands	South Polar Skua (A4ii)	67	Carlini Base		ASPA No. 132	
10	ANT136: Magnetic Island and nearby islands	Princess Elizabeth Land	Adélie Penguin (A4iii)	67	Davis Station			

^1^ Definitions of IBA selection criteria (taken from [3]: Harris et al., 2015). The global (Level A) IBA criteria have been used to identify IBAs in Antarctica. The following definitions of the IBA selection criteria are based on [50]: Fishpool & Evans (2001):

A1: Globally threatened species. “The site is known or thought regularly to hold significant numbers of a globally threatened species, or other species of global conservation concern”. The site qualifies if it is known, estimated or thought to hold a population of a species categorized by the IUCN Red List as Critically Endangered (CR), Endangered (EN) or Vulnerable (VU). In general, the regular presence of a CR or EN species, irrespective of population size, at a site may be sufficient for a site to qualify as an IBA. For VU species, the presence of more than threshold numbers at a site is necessary to trigger selection. The site may also qualify if it holds more than threshold numbers of species in the Near Threatened (NT) category. Thresholds are set regionally, often on a species by species basis. A4: Globally important congregations. A4i: “The site is known or thought to hold, on a regular basis, 1% or more of a biogeographic population of a congregatory waterbird species.” A4ii: “The site is known or thought to hold, on a regular basis, 1% or more of the global population of a congregatory seabird or terrestrial species.” A4iii: “The site is known or thought to hold, on a regular basis, at least 20 000 waterbirds, or at least 10 000 pairs of seabirds, of one or more species.”

Criteria A2, A3 and A4iv are not relevant to the avifauna of Antarctica and so have not been used in this analysis.

^2^ Site Guidelines for Visitors are followed on a voluntary basis

^3^ ASPA: Antarctic Specially Protected Area. ASPAs provide formal legal protection to a site, with entry only permitted in accordance with a permit issued by an appropriate national authority.

^4^ ASMA: Antarctic Specially Managed Area. Entry to an ASMA does not require a permit, but activities should be undertaken in accordance with the ASMA management plan.

**Table 2:** Antarctic IBAs afforded protection under the Antarctic Protected Area system^1^. Footprint score 20–39 (green), 40–59 (yellow), 60–79 (orange), as shown in Fig 3).

No.	Antarctic Important Bird Area	Region	Trigger species (IBA criteria)	Human footprint score	Antarctic Specially Protected Area No.
1	ANT015: Southern Powell Island and adjacent islands	South Orkney Islands	Gentoo Penguin (A1, A4ii), Chinstrap Penguin (A4ii), Imperial Shag (A4i), Southern Giant Petrel (A4ii), Chinstrap Penguin (A4iii)	34	111
2	ANT020: Moe Island	South Orkney Islands	Chinstrap Penguin (A4iii)	36	109
3	ANT046: West Admiralty Bay, King George Island	South Shetland Islands	Gentoo Penguin (A1, A4ii), Adélie, Chinstrap & Gentoo Penguin (A4iii),	58	128
4	ANT047: Potter Peninsula, King George Island	South Shetland Islands	South Polar Skua (A4ii)	67	132
5	ANT048: Ardley Island, King George Island	South Shetland Islands	Gentoo Penguin (A1, A4ii)	75	150
6	ANT049: Harmony Point, Nelson Island	South Shetland Islands	Chinstrap Penguin (A4ii), Snowy Sheathbill (A4ii), Chinstrap Penguin (A4iii)	53	133
7	ANT054: Byers Peninsula, Livingston Island	South Shetland Islands	Antarctic tern (A4i), Kelp Gull (A4i)	49	126
8	ANT081: Cierva Point and offshore islands	Palmer Archipelago / Danco Coast	South Polar Skua (A4ii)	71	134
9	ANT086: Litchfield Island	Palmer Archipelago / Danco Coast	South Polar Skua (A4ii)	64	113
10	ANT095: Avian Island	Marguerite Bay	Adélie Penguin (A1, A4ii), Imperial Shag (A4i), South Polar Skua (A4ii), Adélie Penguin (A4iii)	62	117
11	ANT097: Emperor Island, Dion Islands	Marguerite Bay	Imperial Shag (A4i)	47	107
12	ANT098: Lagotellerie Island	Marguerite Bay	Imperial Shag (A4i)	47	115
13	ANT112: Svarthamaren	Lazarev Sea / Dronning Maud Land	Antarctic Petrel (A4ii), South Polar Skua (A4ii), Antarctic Petrel (A4iii)	49	142
14	ANT119: Taylor Rookery	Mac.Robertson Land	Emperor Penguin (A1, A4ii)	29	101
15	ANT121: Rookery Island	Mac.Robertson Land	Adélie Penguin (A1, A4ii), Adélie Penguin (A4iii)	31	102
16	ANT126: Scullin and Murray Monoliths	Mac.Robertson Land	Adélie Penguin (A1, A4ii)	38	164
17	ANT128: Amanda Bay	Princess Elizabeth Land	Emperor Penguin (A1, A4ii)	49	169
18	ANT141: Haswell Island	Queen Mary Land	Emperor Penguin (A1, A4ii), South Polar Skua (A4ii), Adélie Penguin (A4iii)	38	127
19	ANT145: Ardery Island and Odbert Island	Wilkes Land	Adélie Penguin, Southern Fulmar (A4iii)	53	103
20	ANT147: Clark Peninsula	Wilkes Land	Adélie Penguin (A4iii)	64	136
21	ANT150: Pointe Géologie	Terre Adélie	Emperor Penguin (A1, A4ii)	62	120
22	ANT157: Cape Denison	George V Land	Adélie Penguin (A4iii)	31	162^2^
23	ANT170: Seabee Hook, Cape Hallett	Northern Victoria Land	Adélie Penguin (A1, A4ii)	53	106
24	ANT175: Edmonson Point	Wood Bay / Terra Nova Bay	South Polar Skua (A4ii)	62	165
25	ANT176: Cape Washington	Wood Bay / Terra Nova Bay	Emperor Penguin (A1, A4ii), South Polar Skua (A4ii), Emperor Penguin (A4iii)	49	173
26	ANT186: Caughley Beach, Cape Bird	Ross Island / southern Ross Sea	Adélie Penguin (A1, A4ii), South Polar Skua (A4ii), Adélie Penguin (A4iii)	42	116
27	ANT187: Cape Crozier, Ross Island	Ross Island / southern Ross Sea	Adélie Penguin (A1, A4ii), South Polar Skua (A4ii), Adélie Penguin (A4iii)	47	124
28	ANT188: Beaufort Island	Ross Island / southern Ross Sea	Adélie Penguin (A1, A4ii), South Polar Skua (A4ii), Adélie Penguin (A4iii)	47	105

^1^ IBAs located within Antarctic Specially Managed Areas include: ANT045: Point Hennequin, King George Island, ANT055: Baily Head, Deception Island, ANT056: Vapour Col, Deception Island, ANT085: Cormorant Island, ANT087: Joubin Island, ANT088: Islet south of Gerlache Island.

^2^ ASPA protects primarily historic or terrestrial ecology values at the location, as well as bird life. The ASPAs encompassed by ANT153: Île des Manchots/Empereur Island and ANT165: Cape Adare primarily protects historic values.

**Table 3:** Percentage^1^ of the estimated global population of Antarctic bird species found within IBAs also designated as Antarctic Specially Protected Areas (based upon data contained in [3]: Harris et al, 2015).

Name	Latin name	Red list status	Global population (pairs)^1^	Percentage of estimated global population (pairs) within ASPAs				
				>1%	1–5%	5–10%	10–20%	>20%
Emperor penguin	*Aptenodytes forsteri*	Near threatened	238,000				●	
Gentoo penguin	*Pygoscelis papua*	Near threatened	387,000			●		
Chinstrap penguin	*Pygoscelis antarctica*	Least concern	2,666,667					●
Adélie penguin	*Pygoscelis adeliae*	Near threatened	3,790,000				●	
Macaroni penguin	*Eudyptes chrysolophus*	Vulnerable	6,300,000			^2^		
Southern giant petrel	*Macronectes giganteus*	Least concern	50,000		●			
Antarctic petrel	*Thalassoica antarctica*	Least concern	3–7,000,000			○	○	
Cape petrel	*Daption capense*	Least concern	670,000	●				
Snow petrel	*Pagodroma nivea*	Least concern	1,300,000		●			
Southern fulmar	*Fulmarus glacialoides*	Least concern	1,000,000	●				
Antarctic prion	*Pachyptila desolata*	Least concern	16,600,000			^2^		
Wilson’s storm-petrel	*Oceanites oceanicus*	Least concern	4–10,000,000	●				
Black-bellied storm-petrel	*Fregetta tropica*	Least concern	160,000			^2^		
Imperial (Antarctic) shag	*Phalacrocorax* [*atriceps*] *bransfieldensis*	Least concern	13,333				●	
Brown skua	*Catharacta antarctica*	Least concern	3–7500			^2^		
South polar skua	*Catharacta maccormicki*	Least concern	3–7500					●
Kelp gull	*Larus dominicanus*	Least concern	10–20,000		○	○		
Antarctic tern	*Sterna vittata*	Least concern	36,666			●		
Snowy (greater) sheathbill	*Chionis albus*	Least concern	10,000		●			

● Percentage value is within the range indicated

○ Global bird populations (pairs) are not accurately known for all species (see column 4). Where the possible percentage population within ASPAs may be within two percentage ranges, both are indicated with this symbol.

^1^ Percentages are likely to be conservative estimates, as data for each species within all ASPAs were not available. This may be particularly true for species with colonies found in remote locations and not subject to regular counts. Values are derived from counts of bird pairs rather than individuals (see [3]: Harris et al., 2015, pg. 4). Smaller numbers or lower concentrations of bird species are also likely to breed within other ASPAs not designated as IBAs.

^2^ Species recorded and possibly breeding within at least one ASPA, but numbers are not available.

## Discussion

This study completes the mapping of global human footprint by producing the first continent-wide map for terrestrial Antarctica [21, 22]. At a continent-wide scale human footprint was much lower than most other areas of the Earth. However, at a regional spatial scale, footprint was often correlated with accessibility to ice-free land by sea. For example, in the Ross Sea region the accessible coastline hosts several facilities and human footprint values are correspondingly high, while the values for the remote, uninhabited and largely land-locked Transantarctic Mountains were comparatively low. In some areas, ice-free coastal sites of large extent were often found to be subject to greater human activity than those of smaller extent. For example, on the South Shetland Islands, most major ice-free promontories were sites of substantial human activity or infrastructure (Fig 2). This may indicate that the availability of ice-free ground for further human colonization at some locations may be reaching a limit, and notably on the northern Antarctic Peninsula and offshore islands. Importantly, this means that substantial amounts of ground free of direct human activity and associated impacts (including wildlife disturbance and habitat destruction) are no longer available to indigenous flora and fauna. The designation of Antarctic Specially Protected Areas (ASPAs) generally reduced the human footprint values of the selected areas, as entry to these sites is conditional upon visitors obtaining permits, which are generally only allocated for scientific or environmental management purposes. Nevertheless, ASPAs close to existing research stations often had relatively high footprint values, as access by scientific staff was more readily achievable (Hughes et al. 2013).

It should be highlighted that the generated maps (e.g. Figs 1 & 2) indicate pressure, but do not account for the degree of site vulnerability or resilience. Furthermore, the footprint model incorporates data relevant to the current distribution of infrastructure and human activity; however, the addition of a temporal element to the model may allow insight into cumulative impacts at sites or across the continent as a whole (Hughes et al., 2011). Thus, a limitation of the study is the capacity to detect the exact levels of disturbance experienced by Antarctic terrestrial species at the most vulnerable stages of their life cycles (such breeding or moulting). However, since most human activities in Antarctica peak in the austral summer period considerable interference could be expected.

Moreover, the picture of human footprint presented here is generated from information on present activities (2015–16) and does not reflect the cumulative historical occupation of the sites. Therefore, it should be viewed as an indication of the current pressures to Antarctic ecosystems, and therefore subjected to changes with time.

### Use of footprint information in management of human activities

**Fig 5:**
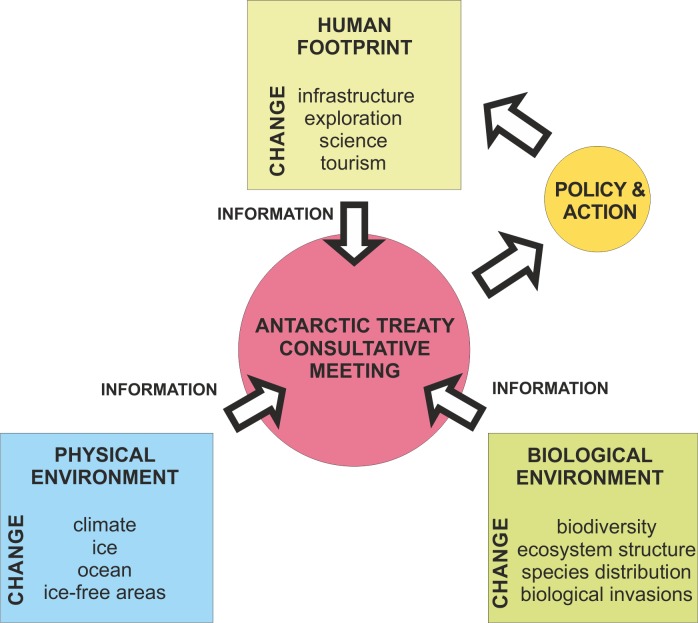
Summary of information necessary to facilitate evidence-based environmental management decisions by Antarctic Treaty Consultative Meeting policy-makers. The production of a human footprint mapping tool fills a gap in the information readily available to policy-makers.

Climate change and expanding human footprint are having an increasing impact upon Antarctic terrestrial ecosystems and their synergistic action may increase conservation challenges across the continent and beyond [1, 2, 35]. It is the responsibility of the Antarctic Treaty Consultative Meeting to ensure the agreed principles within the Protocol on Environmental Protection to the Antarctic Treaty are applied, taking into consideration specialist advice provided by the Committee for Environmental Protection. The Protocol states that ‘*activities in the Antarctic Treaty area shall be planned and conducted so as to limit adverse impacts on the Antarctic environment…*’. However, until recently, spatial and temporal information on the three major components that are needed to assist Parties in their policy and environmental management decisions were not readily accessible to policy makers, i.e. information on (1) the Antarctic physical environment, (2) the biological environment and (3) the extent of human footprint (see Fig 5).

This situation improved with the development of spatial management tools. Morgan et al., (2007) developed the Environmental Domains Analysis (EDA), which categorised Antarctica into 21 domains based upon physical parameters such as climate, topography and geology. Building on the EDA framework [44] the study of Terauds and colleagues [45] drew upon expert opinion and available biodiversity data to divide Antarctica into 15 biologically distinct biogeographical regions, named Antarctic Conservation Biogeographic Regions (ACBRs). In this study we have provided a management tool to describe the third component, i.e. Antarctic human footprint. Combined with these other management tools, footprint mapping has the potential to assist the ATCM in determining areas at risk of non-native species introductions and areas at risk of transfer of indigenous species between biogeographic regions leading to biological homogenisation [4, 10, 36, 46]. Footprint mapping may also be useful in the development of the Antarctic Protected Area system, which has received criticism for being inadequate and unrepresentative [11, 37]. In particular, unimpacted locations may be identified for designation as Antarctic Specially Protected Areas that are to be conserved as reference sites, or even maintained as unvisited ‘inviolate’ locations [28, 33]. Alternatively, footprint maps may highlight areas of intense human activity where the creation of Antarctic Specially Managed Areas (ASMAs) may be important to avoid conflict and facilitate cooperation between stakeholders, and ensure adequate protection, rather than further displacement of biological communities and wildlife. It may also be useful in planning monitoring programmes for potential human impact including pollutants, environmental damage and non-native species [5, 9, 47].

### Antarctic important bird areas

To demonstrate the potential use of the human footprint model, the potential levels of human pressure on Antarctica’s 204 Important Bird Areas were assessed by determining the footprint score for each area. The data showed that IBAs with the highest footprint scores were located in the northern Antarctic Peninsula region and close to research stations in coastal locations of East Antarctica and the Ross Sea region (see Fig 3 and Table 1).

An IBA can be designated as an ASPA to protect a site for scientific research or for conservation purposes, including minimizing human impact at the site. However, less than 14% of IBAs have been designated as ASPAs (see Table 2 and Fig 4). Furthermore, of the IBAs most vulnerable to human impact, 73% remain without legally binding protection through ASPA designation (i.e. the 30 IBAs with footprint scores of over 60) (Table 2). Of greater concern still, of the four IBAs with the highest human footprint scores, none have been designated as ASPAs nor given additional protection within any Antarctic Specially Managed Areas. More specifically, IBA No. ANT074 Hope Bay, Trinity Peninsula, had by far the highest footprint score (97). The c. 125,000 Adélie penguins and other bird species within this IBA are located immediately adjacent to two research stations, one of which is a local hub for helicopter air operations. No internationally recognized management has been agreed for this location, despite visitation levels of up to 3000 visitors per year. Research station managers may impose restrictions on personnel residing at the stations regarding access to nearby IBAs, but their jurisdiction may not extends to other visitors.

Several IBAs at low risk of human activity and potential impact have received protection through ASPA designation. The justification for these designations over other IBAs is not always clear, but may be to protect exceptional assemblages of avifauna or to facilitate scientific investigations (e.g. IBA No. ANT015/ASPA No. 111 Southern Powell Island and adjacent islands, South Orkney Islands, and IBA No. ANT119/ASPA No. 101 Taylor Rookery, Mac.Robertson Land).

Antarctic Specially Managed Areas (ASMAs) are designated to assist in the planning and co-ordination of activities, avoid possible conflicts, improve co-operation between Parties or minimise environmental impacts. Although the measures set out in their management plans are hortatory, Restricted Zones can be designated, where human access may only be allowed under special circumstances, as has been done at ANT085: Cormorant Island that lies within ASMA No. 7 Southwest Anvers Island and Palmer Basin. However, the additional protection afforded by designation of Scientific Zones within ASMAs may not always be clear (e.g. those within ASMA No. 1 Admiralty Bay, King George Island). Fildes Peninsula (King George Island) does not possess an ASMA designation despite the high levels of footprint present [13]. Designation of an ASMA could be supported by the existence of IBA No. ANT145 which lies within the boundaries of ASPA 150 Ardley Island and receives one of the highest numbers of permitted visitors in Antarctica (see [26]).

IBAs with some of the highest footprint scores are located in the northern Antarctic Peninsula. Some IBAs experience high visitation from tourists (up to 22,000 visitors per year) and have site management provided by non-mandatory ‘Site Guideline for Visitors’ (available at: http://www.ats.aq/e/ats_other_siteguidelines.htm) (e.g. IBA No. ANT089 Petermann Island, Graham Coast, IBA No. ANT083 Cuverville Island, Palmer Archipelago/Danco Coast, and IBA No. ANT073 Brown Bluff, Trinity Peninsula; see Table 1). However, the effectiveness of ‘Site Guidelines for Visitors’ is open to question. Site Guidelines may limit the number of visitors allowed ashore at any one time, generally restrict approach distance to wildlife to a minimum of 5 m, and may describe ‘closed areas’ but do not prohibit movement amongst bird colonies outside these areas. Due to the hortatory nature of these guidelines, the consequences of breaching the guidelines are not clear. Coetze and Chown [48], following a recent meta-analysis of human disturbance impact on Antarctic wildlife, recommended that management guidelines for different species found at different locations be developed on a case-by-case basis. As little or no wildlife disturbance research has been undertaken at most visitor sites, the credibility of wildlife minimum approach distance recommendations within existing Site Guideline for Visitors is therefore in doubt. Furthermore, the ATCM’s ‘*General Guidelines for Visitors to the Antarctic*’ (available at: http://www.ats.aq/documents/recatt/Att483_e.pdf) are also of limited use with their instruction to ‘*Maintain an appropriate distance from wildlife*. *While in many cases a greater distance may be appropriate*, *in general don’t approach closer than 5 m*’.

Examination of the effectiveness of the current ASPA system in affording legal protection to Antarctica’s different bird species populations, revealed great variability in the percentage of estimated bird populations protected within ASPAs, albeit the availability of data made this assessment difficult or impossible for some species (Table 3). While formal protection was high for some species, smaller species were often less well protected with less than 1% of the global breeding population found within ASPAs. For other species inadequate data meant estimates were not possible pointing to a need for improved bird population monitoring. Most Antarctic bird species have been evaluated as either Least Concern or Near Threatened under the IUCN categories, so enhanced protection may not be a priority in terms of global populations. Nevertheless, it could be argued that higher conservation standards exist for Antarctica: under the Protocol, the Parties designated Antarctica as a ‘*natural reserve*’ and commit themselves to the comprehensive protection of the Antarctic environment. The Protocol also states that ASPA designation is appropriate for ‘*major colonies of breeding native birds or mammals*’ (Annex V, Article 3(2c)), but to date several authors have suggested that the systematic implementation of area protection has been lacking [2, 11, 33, 34, 49]. To inform the selection of candidate sites for future ASPA designation an assessment of two IBA characteristics may be useful, i.e. (1) the potential vulnerability of existing IBAs to human activity, and (2) the number and diversity of species they contain may. The footprint data provided in this paper fulfils the first of these criteria and the data in Table 3 alongside the work of Harris and colleagues [3] fulfills the second. This information combined with local knowledge of specific IBAs creates the opportunity for more comprehensive use of the Antarctic protected area system in protection of the continent’s avifauna.

## Conclusions

Taking a broad view, Antarctica is subject to less human activity that most other areas of the Earth; however, at a finer spatial scale many areas of scare coastal ice-free ground are under increasing pressure from human activities and substantial human impacts have been recorded including habitat destruction and disturbance of wildlife (e.g. King George Island, South Shetland Island) [1, 5, 13]. Until now, comprehensive models of human footprint within the continent have not been available to inform environmental policy and management decisions. Application of the footprint model to assess the use of existing Antarctic spatial management tools for avifauna conservation showed designation of protected areas to be unsystematic, in some cases leaving penguin and flying bird species without formal legal protection in some of the Important Bird Areas most potentially vulnerable to human activity and impact. It is hoped that the human footprint map generated here, alongside other available management tools, may prove useful for policy-makers in their work on issues including avifauna protection, environmental impact assessment, environmental monitoring, non-native species, area protection and, ultimately, a wider Antarctic conservation strategy [2, 34, 50]. Spatial footprint data generated here are available on the Dryad Digital Repository: http://dx.doi.org/10.5061/dryad.fp0nh.

## Supporting Information

S1 Table Scoring of land use levels in Antarctica. (DOCX)

S2 Table Scoring of human density levels in Antarctica. (DOCX)

S3 Table Scoring of accessibility levels in Antarctica. (DOCX)

S4 Table Score of human footprint levels calculated for major stations on ice. (DOCX)
