# Correction: High Resolution Spatial Mapping of Human Footprint across Antarctica and Its Implications for the Strategic Conservation of Avifauna

**DOI:** 10.1371/journal.pone.0173649

**Published:** 2017-03-03

**Authors:** Luis R. Pertierra, Kevin A. Hughes, Greta C. Vega, Miguel Á. Olalla-Tárraga

Table 3 contains an error in the estimation of the number of global population (pairs) of Chinstrap penguins (*Pygoscelis antarctica*) within ASPAs. It should read as 5–10%. Please see the corrected Table 3 here.

**Table 3:** Percentage^1^ of the estimated global population of Antarctic bird species found within IBAs also designated as Antarctic Specially Protected Areas (based upon data contained in [3]: Harris et al, 2015).

Name	Latin name	Red list status	Global population (pairs)^1^	Percentage of estimated global population (pairs) within ASPAs				
				>1%	1–5%	5–10%	10–20%	>20%
Emperor penguin	*Aptenodytes forsteri*	Near threatened	238,000				●	
Gentoo penguin	*Pygoscelis papua*	Near threatened	387,000			●		
Chinstrap penguin	*Pygoscelis antarctica*	Least concern	2,666,667			●		
Adélie penguin	*Pygoscelis adeliae*	Near threatened	3,790,000				●	
Macaroni penguin	*Eudyptes chrysolophus*	Vulnerable	6,300,000			^2^		
Southern giant petrel	*Macronectes giganteus*	Least concern	50,000		●			
Antarctic petrel	*Thalassoica antarctica*	Least concern	3–7,000,000			○	○	
Cape petrel	*Daption capense*	Least concern	670,000	●				
Snow petrel	*Pagodroma nivea*	Least concern	1,300,000		●			
Southern fulmar	*Fulmarus glacialoides*	Least concern	1,000,000	●				
Antarctic prion	*Pachyptila desolata*	Least concern	16,600,000			^2^		
Wilson’s storm-petrel	*Oceanites oceanicus*	Least concern	4–10,000,000	●				
Black-bellied storm-petrel	*Fregetta tropica*	Least concern	160,000			^2^		
Imperial (Antarctic) shag	*Phalacrocorax* [*atriceps*] *bransfieldensis*	Least concern	13,333				●	
Brown skua	*Catharacta antarctica*	Least concern	3–7500			^2^		
South polar skua	*Catharacta maccormicki*	Least concern	3–7500					●
Kelp gull	*Larus dominicanus*	Least concern	10–20,000		○	○		
Antarctic tern	*Sterna vittata*	Least concern	36,666			●		
Snowy (greater) sheathbill	*Chionis albus*	Least concern	10,000		●			

● Percentage value is within the range indicated

○ Global bird populations (pairs) are not accurately known for all species (see column 4). Where the possible percentage population within ASPAs may be within two percentage ranges, both are indicated with this symbol.

^1^ Percentages are likely to be conservative estimates, as data for each species within all ASPAs were not available. This may be particularly true for species with colonies found in remote locations and not subject to regular counts. Values are derived from counts of bird pairs rather than individuals (see [3]: Harris et al., 2015, pg. 4). Smaller numbers or lower concentrations of bird species are also likely to breed within other ASPAs not designated as IBAs.

^2^ Species recorded and possibly breeding within at least one ASPA, but numbers are not available.
