# Supplementary material for: High Resolution Spatial Mapping of Human Footprint across Antarctica and Its Implications for the Strategic Conservation of Avifauna
Source: PLoS One. 2017 Jan 13;12(1):e0168280. doi: 10.1371/journal.pone.0168280 (PMC5235374; doi:10.1371/journal.pone.0168280)
Supplement: S1 Table — (DOCX) [file pone.0168280.s001.docx]

Supplementary Table 1. Land uses in Antarctica

| No. | Land use feature | Description | Area (radius) | Score |
| --- | --- | --- | --- | --- |
| 1 | Large built-up environment | Scientific stations, | 0.5 km* | 10 |
| 2 | Visitor site | landing site centroid | 0.5 km | 9 |
| 3 | Small built-up environment | Camps, depots, aerodromes, stations | 0.5 km | 8 |
| 4 | Major built-up influence | Station surroundings | 5 km | 6 |
| 5 | Visitor landing site - minor influence | Coastal visitor landing site surroundings | 2.5 km | 5 |
| 6 | ASPA designation | Protected areas | Variable | 3 |
| 7 | Uncategorized | Areas with no reported activity | Variable | 1 |
