# Supplementary material for: High Resolution Spatial Mapping of Human Footprint across Antarctica and Its Implications for the Strategic Conservation of Avifauna
Source: PLoS One. 2017 Jan 13;12(1):e0168280. doi: 10.1371/journal.pone.0168280 (PMC5235374; doi:10.1371/journal.pone.0168280)
Supplement: S2 Table — (DOCX) [file pone.0168280.s002.docx]

Supplementary Table 2 Human densities

| No. | Density | Score |
| --- | --- | --- |
| 1 | 10 people km^-2^ y^-1^ | 10 |
| 2 | 9 people km^-2^ y^-1^ | 9 |
| 3 | 8 people km^-2^ y^-1^ | 8 |
| 4 | 7 people km^-2^ y^-1^ | 7 |
| 5 | 6 people km^-2^ y^-1^ | 6 |
| 6 | 5 people km^-2^ y^-1^ | 5 |
| 7 | 4 people km^-2^ y^-1^ | 4 |
| 8 | 3 people km^-2^ y^-1^ | 3 |
| 9 | 2 people km^-2^ y^-1^ | 2 |
| 10 | 1 person km^-2^ y^-1^ | 1 |
