# Supplementary material for: High Resolution Spatial Mapping of Human Footprint across Antarctica and Its Implications for the Strategic Conservation of Avifauna
Source: PLoS One. 2017 Jan 13;12(1):e0168280. doi: 10.1371/journal.pone.0168280 (PMC5235374; doi:10.1371/journal.pone.0168280)
Supplement: S3 Table — (DOCX) [file pone.0168280.s003.docx]

Supplementary Table 3. Accessibility

| No. | Walking distance (km)  from coast or station | Aircraft flying distance (km) | Score |
| --- | --- | --- | --- |
| 1 | Less than 1 | 50 | 10 |
| 2 | 1 to 2 | 100 | 9 |
| 3 | 2 to 4 | 150 | 8 |
| 4 | 4 to 8 | 200 | 7 |
| 5 | 8 to 16 | 250 | 6 |
| 6 | 16 to 32 | 300 | 5 |
| 7 | 32 to 64 | 350 | 4 |
| 8 | 64 to 128 | 400 | 3 |
| 9 | 128 to 256 | 450 | 2 |
| 10 | 256 or more | 450 or more | 1 |
