# Supplementary material for: High Resolution Spatial Mapping of Human Footprint across Antarctica and Its Implications for the Strategic Conservation of Avifauna
Source: PLoS One. 2017 Jan 13;12(1):e0168280. doi: 10.1371/journal.pone.0168280 (PMC5235374; doi:10.1371/journal.pone.0168280)
Supplement: S4 Table — (DOCX) [file pone.0168280.s004.docx]

Supplementary Table 4. Stations on Permanent Ice

| Name | Status | | Equivalent Score |
| --- | --- | --- | --- |
| Amudsen-Scott South Pole Station | | Year-round | 82 |
| Halley Station | | Year-round | 82 |
| Neumayer Station III | | Year-round | 82 |
| Concordia Station | | Year-round | 82 |
| Vostok Station | | Year-round | 82 |
| Taishan Station | | Seasonal | 82 |
| Kohnen Station | | Seasonal | 71 |
| Kunlun Station | | Seasonal | 69 |
| Dome Fuji Station | | Seasonal | 67 |
|  |  | |  |
